# Strategies for working across Canadian practice-based research and learning networks (PBRLNs) in primary care: focus on frailty

**DOI:** 10.1186/s12875-021-01573-y

**Published:** 2021-11-12

**Authors:** Manpreet Thandi, Sabrina T. Wong, Sylvia Aponte-Hao, Mathew Grandy, Dee Mangin, Alexander Singer, Tyler Williamson

**Affiliations:** 1 Centre for Health Services and Policy Research & School of Nursing, University of British Columbia, 201-2206 East Mall, Vancouver, BC V6T IZ3 Canada; 2 Centre for Health Services and Policy Research & School of Nursing, University of British Columbia, 2211 Wesbrook Mall, Vancouver, BC V6T 2B5 Canada; 3 Department of Community Health Sciences, Cumming School of Medicine, University of Calgary, 3280 Hospital Drive NW, Calgary, Alberta T2N 2Z6 Canada; 4 Department of Family Medicine, Dalhousie University, 1465 Brenton Street, Suite 402, Halifax, Nova Scotia B3J 3T4 Canada; 5 Department of Family Medicine, McMaster University, 1280 Main St W, Hamilton, ON L8S 4L8 Canada; 6 Department of Family Medicine, University of Manitoba, D009-780 Bannatyne Ave, Winnipeg, MB R3E 0W2 Canada; 7 Centre for Health Informatics & Department of Community Health Sciences, Cumming School of Medicine, University of Calgary, 3280 Hospital Drive NW, Calgary, Alberta T2N 2Z6 Canada

**Keywords:** Practice-based research and learning networks, Learning health systems, Primary care, Frailty, Multi-jurisdictional collaboration

## Abstract

**Background:**

Practice based research and learning networks (PBRLNs) are groups of learning communities that focus on improving delivery and quality of care. Accurate data from primary care electronic medical records (EMRs) is crucial in forming the backbone for PBRLNs. The purpose of this work is to**:** (1) report on descriptive findings from recent frailty work, (2) describe strategies for working across PBRLNs in primary care, and (3) provide lessons learned for engaging PBRLNs.

**Methods:**

We carried out a participatory based descriptive study that engaged five different PBRLNs. We collected Clinical Frailty Scale scores from a sample of participating physicians within each PBRLN. Descriptive statistics were used to analyze frailty scores and patients’ associated risk factors and demographics. We used the Consolidated Framework for Implementation Research to inform thematic analysis of qualitative data (meeting minutes, notes, and conversations with co-investigators of each network) in recognizing challenges of working across networks.

**Results:**

One hundred nine physicians participated in collecting CFS scores across the five provinces (*n* = 5466). Percentages of frail (11-17%) and not frail (82-91%) patients were similar in all networks, except Ontario who had a higher percentage of frail patients (25%). The majority of frail patients were female (65%) and had a significantly higher prevalence of hypertension, dementia, and depression. Frail patients had more prescribed medications and numbers of healthcare encounters. There were several noteworthy challenges experienced throughout the research process related to differences across provinces in the areas of: numbers of stakeholders/staff involved and thus levels of burden, recruitment strategies, data collection strategies, enhancing engagement, and timelines.

**Discussion:**

Lessons learned throughout this multi-jurisdictional work included: the need for continuity in ethics, regular team meetings, enhancing levels of engagement with stakeholders, the need for structural support and recognizing differences in data sharing across provinces.

**Conclusion:**

The differences noted across CPCSSN networks in our frailty study highlight the challenges of multi-jurisdictional work across provinces and the need for consistent and collaborative healthcare planning efforts.

## Background

Significant resources have been invested by healthcare organizations in integrating comprehensive electronic medical records (EMRs) into clinical care [1]. EMRs have led to significant improvements in the digitization of healthcare, allowing for clinical information to support quality improvement and research initiatives, which have facilitated the development of learning health systems [1]. Learning health systems (LHSs) are organizations where “science, informatics, incentives, and culture are aligned for continuous improvement and innovation” ([2], p.252). LHSs have been defined by the Institute of Medicine as a vision for an integrated health system that generates new knowledge as an ongoing, natural by-product of the care experience while refining and delivering best practices for continuous health and healthcare improvement [3, 4]. In order for LHSs to be achieved they must as Friedman et al. [5] stated, “harness the power of data and analytics to learn from every patient, and feed the knowledge of ‘what works best’ back to clinicians, public health professionals, patients, and other stakeholders to create cycles of continuous improvement” (p.44). LHSs support learning as a product of everyday care, made possible through infrastructure that enables high-quality clinical data to be collected, analysed, and acted upon [6].

LHSs can also be thought of as common infrastructure and governance structures with shared values and incentives [5]. Primary care providers can make a significant contribution to LHSs. Primary care providers are typically the first point of contact to healthcare for patients; they focus on prevention, coordination, stewardship, and managing chronic complex illnesses [2]. Thus, through partnerships between providers, researchers, and educators, primary care providers can improve the adoption of LHSs [2].

Primary care practice-based research and learning networks (PBRLNs) can be considered a key component in achieving high performing LHSs. PBRLNs are considered groups of learning communities of care practice since they focus on improving delivery and quality of care [7]. PBRLNs in primary care can improve efficiency, reduce variation across systems, and improve the quality and safety of care [8], while recognizing the valuable input of key stakeholders, including patients, policy makers, and clinicians. PBRLNs draw on the experiences and insights of experts to improve the practice of primary care [9] and support quality improvement activities within primary care to adopt an evidence-based culture. In order to enhance the quality of primary care practice, values and goals across PBRLNs must align. Accurate data from primary care EMRs are crucial to form the backbone for PBRLNs and LHSs. Powered by analytics, big data, and information exchange, EMRs have the potential to significantly improve health and healthcare when they are used to their full potential [2].

Previous research involving PBRLNs demonstrates that team-based research is important to address complex health problems and necessary if primary care is to improve adoption, implementation, and sustainability of clinical practice [10]. Dania et al.’s [11] recent scoping review reports on 229 publications that describe the establishment of 93 PBRNs in 15 countries. Their review includes articles that refer to research projects conducted by PBRNs that also provide information about their establishment; the authors report on key PBRN activities and themes. Of these 229 publications, seven [12–18] reflected a Canadian context. To our knowledge, our paper is the first to report on frailty detection using PBRLNs across multiple provinces in Canada.

Primary care serves two functions in the Canadian primary health care system: (1) to provide direct first contact services by family physicians and nurse practitioners, and (2) to coordinate continuity of care across the health care system so care remains integrated when Canadians need more specific and specialized services [19]. Responding to community needs is a key element of primary health care in Canada and includes services such as prevention, treatment, and management of diseases and injuries; basic emergency services; referrals and coordination with other levels of health care such as hospitals and specialized care; mental health care; palliative and end of life care; and health promotion [19]. Primary care thus refers to the first line of clinical services that provide an entry point into the health care system [20]. It refers to the delivery of community-based health care services and provides coordination of care to enable access to other health care services and providers [21].

Frailty is a significant concern in primary care practice. PBRLNs and LHSs are poised to enact solutions that can mitigate primary care provider’s challenges in managing frailty. The Canadian Frailty Network [22] defines frailty as a condition of reduced function and health in which older adults living with frailty are at an increased risk of health decline and negative health-related outcomes. In primary care, the goals of caring for those who are frail are to: prevent or delay increasing frailty severity, improve function and quality of life, and to avoid unnecessary admission to hospital or long-term care [23, 24]. There is currently no standardized way to identify and manage frailty in primary care in Canada; however, recent PBRLN work has proposed a frailty case definition for use in primary care EMRs [25].

The purposes of this paper are to: (1) report on descriptive findings from recent frailty work, (2) describe strategies for working across practice-based research and learning networks (PBRLNs) in primary care, and (3) provide lessons learned for engaging PBRLNs.

## Methods

### Study design and setting

We carried out a participatory based descriptive study with the Canadian Primary Care Sentinel Surveillance Network (CPCSSN). CPCSSN (www.cpcssn.ca) is a pan-Canadian network made up of practice-based research and learning networks [26]. Most regional networks are provincial with the exception of Ontario which consists of six regional networks and Alberta which has two networks. Across CPCSSN there are > 1500 participating family physicians, nurse practitioners and their staff and > 2 million patients who participate.

The CPCSSN has successfully built trusting relationships in its PBRLNs between primary care clinicians and researchers over the past 12 years. It extracts EMR data from consenting clinicians, standardizes the data into a common schema based on internationally accepted ontologies and terminologies and makes the data available for purposes of quality improvement, communicable and non-communicable disease surveillance and research [27]. Participating clinicians are provided reports on their data that compares them to their participating site colleagues and the rest of their PBRLN through CPCSSN’s data presentation tool [28]. CPCSSN has also developed processes that allow participating clinics to securely re-identify and view their own data to enable them to prepare customized lists of patients in specific risk populations [29].

For the purposes of this work, we were interested in creating a reference dataset from which we tested supervised machine learning techniques to derive and validate a case definition on frailty [25]. Supervised machine learning is a type of machine learning that learns by example, where algorithms ‘learn’ how to classify or predict new observations based on previously seen data. The quality of the data used for training machine learning algorithms directly relates to the quality of the algorithms that can be produced and are sometimes referred to as the reference standard. In medical research, supervised machine learning is often used to classify whether a patient has a certain disease based on patient history and past medical charts. The reference standard in this case would contain accurate frailty diagnoses, paired with all known patient information extracted from EMRs. The comprehensive methods and list of algorithms used for machine learning analysis are reported elsewhere [25], but include classification and regression tree, logistic regression, support vector machines, gradient boosted machines, and neural networks.

Previous work using supervised machine learning (classification and regression tree) for the identification of frailty in EMR data by Williamson et al. [23] used data from Alberta, Canada. Tarekegn et al. [30] used Italian administrative health records to develop predictive models for frailty conditions (mortality, urgent hospitalization, disability, fracture, and emergency admission) using neural networks, genetic programming, support vector machines, random forest, logistic regression, and decision tree. The authors found that neural networks showed high performance in predicting mortality and that support vector machines showed high performance in predicting urgent hospitalization. In our study, gradient boosted machines achieved the highest sensitivity (78.14%) and specificity (74.41%) in predicting frailty. Other research has been completed using machine learning methods to classify frailty, but frailty was defined using other instruments such as the Clinical Frailty Scale [23], the Frailty Phenotype [31], or the electronic frailty index [32]. Our study is the first to use pan-Canadian primary care EMR data to create a frailty case definition using machine learning.

In our study, five CPCSSN networks participated in data collection and building the reference set of frail patients: British Columbia (BC), Alberta (AB) (*n* = 1 network, SAPCReN in Calgary), Manitoba (MB), Ontario (ON) (*n* = 1 network, MUSIC in Hamilton), and Nova Scotia (NS). Quebec, New Brunswick, and Newfoundland/Labrador were not included within the scope of this study due to funding. Future studies would benefit from including these provinces. Saskatchewan, the Northwest Territories, and the Yukon do not have regional CPCSSN networks at this time and thus were not included in this study. The methods used to create the reference dataset are described here.

### Outcome of interest: frailty assessment tool

The determination of frailty in our study is based on the Rockwood Clinical Frailty Scale (CFS). Primary care clinicians used the Rockwood CFS (Appendix 1) to classify the current degree of frailty observed in a randomly selected subset of their patients. While there are more comprehensive methods to assess frailty, such as the Comprehensive Geriatric Assessment (CGA) scale, they typically take up to  one hour or more to complete. The CGA was discussed as the gold standard to identify patients with frailty, but it was unrealistic to conduct this assessment on a large number of patients due to the extensive time required.

The CFS is a one-page document, taking about 30 seconds to one minute per patient for the clinician to complete. It is simple, efficient, and easy to use in addition to being be a valid and clinically important tool [33, 34]. Patients also do not need to come into clinics to be assessed and there is no associated cost to using the tool. The Rockwood CFS is one of the most widely accepted tools used to identify frailty in Canada. It uses clinical judgement to assign a score from 1 (very fit) to 9 (terminally ill) [33, 34]. The CFS has demonstrated high interrater reliability between physicians and multidisciplinary teams (ICC = 0.97, *p* <  0.001). It also demonstrates convergent validity in being highly correlated (*r* = 0.80) with other established frailty tools [33, 34] and has demonstrated predictive validity for mortality rates and entry into institutions [33].

### Procedures

After obtaining consent, clinicians provided assessments of frailty using the Rockwood CFS on a randomly selected subset of their patients who were over the age of 65 and who had had a clinic visit within the last 24 months. Our goal was to collect a reference dataset of 900 CFS scores per network (*n* = 4500 total) in order to carry out our supervised machine learning analyses, which are reported elsewhere [25].

Each participant completed between 15 and 180 patient assessments using the CFS, depending on how many clinicians were recruited in each network. Lists of eligible patients were either generated by research team members through an EMR search query of eligible patients or clinicians performed their own search query. Clinicians were provided with a list (either a complete list of their patient panel, or a subset) of their patients who met the eligibility criteria. They either completed the assessments on their own, scheduled time with a research team member (author MT), or had a PBRLN working party (MUSIC). Once clinicians completed their assessments, patient IDs were matched to CPCSSN IDs. CPCSSN IDs and their associated CFS scores were recorded on spreadsheets, ensuring no personal patient information was ever taken out of the clinics.

EMR data for all of the patients with a CFS score was compiled into one dataset. All procedures and analyses were approved by regional network directors’ ethics boards, including: University of British Columbia, University of Calgary, University of Manitoba, McMaster University, and Dalhousie University.

Data for this work were also derived from qualitative sources including study team meeting minutes and email exchanges with the study team members. All notes were electronically documented.

### Analysis

Descriptive statistics were used to analyze CFS scores and associated frailty risk factors and demographics. We did not investigate the relationship between variables and the presence of frailty in this work. Future work would benefit from such multivariable analyses. Certain associations between variables of importance and frailty are reported elsewhere [25].

We used the Consolidated Framework for Implementation Research (CFIR) to guide analyses of the qualitative data since conducting a widespread effort to gather CFS is much like implementing an intervention. The CFIR (Appendix 2) is a conceptual framework developed to guide assessment of intervention implementation in a systematic way; it helps to identify factors that might potentially influence intervention implementation and effectiveness [35, 36]. The CFIR is composed of five domains including: 1) intervention characteristics, or features of the intervention; 2) inner setting, or features of the implementing organization; 3) outer setting, or features of the external environment; 4) characteristics of individuals involved; and, 5) implementation process, including strategies that might influence implementation [36]. Thematic analysis of meeting minutes, notes, and conversations with co-investigators of each network reflecting various aspects of the research process was informed by the CFIR.

## Results

### Recruitment of family physicians (FPs)

Table 1 displays results related to the recruitment of FPs. One hundred nine FPs participated in collecting CFS scores across the five provinces. Most of the FPs were recruited through existing relationships with CPCSSN (*n* = 100), while the remaining were recruited through snowball sampling with support from initial participants (*n* = 9). There were multiple stakeholders involved in the recruitment process including: CPCSSN regional network directors for each province, research assistants and coordinators, medical office assistants, data managers, and existing physician participants who supported snowball sampling. In Alberta, the head of the Department of Family Medicine committed to involvement for his entire department and provided in-kind support staff to facilitate collection of the CFS scores.

**Table 1:** Recruitment of Family Physicians

Network (*n* = FPs recruited)	Recruitment Strategies	Stakeholders Involved in Recruitment
British Columbia (BC) (*n* = 5 in 3 separate clinics)	Pre-existing relationships (*n* = 2) Snowball sampling with support from initial participants (*n* = 3) Because not all recruited FPs were existing CPCSSN sentinels, FPs needed to first consent to join CPCSSN, and then consent to participate in the frailty study.	CPCSSN regional network director Research assistant Medical office assistants Existing physician participants
Alberta (AB) (*n* = 52 from the Department of Family Medicine at 3 sites)	The Department of Family Medicine at the University of Calgary was very interested in the work and thus the FPs from the department were the participants.	CPCSSN regional network director Department of Family Medicine (Head of department and administrative personnel)
Manitoba (MB) (*n* = 10 in 3 separate clinics)	Pre-existing relationships (*n* = 3) Snowball sampling with support from initial participants (*n* = 7) Although all three clinics were part of CPCSSN, there were two new providers that consented to join CPCSSN and subsequently participate in the frailty study.	CPCSSN regional network director Research coordinator Existing physician participants
Nova Scotia (NS) (*n* = 5 in 5 separate clinics)	Existing CPCSSN FPs (via email)	CPCSSN regional network director Data manager Existing physician participants
Ontario (ON) *n* = 37 in a Family Health Team	Existing MUSIC FPs (in person, written material)	CPCSSN regional network director Research assistant Existing physician participants

### Data collection strategies

Table 2 displays strategies used to collect data in the 5 provinces. In working with the clinicians who provided the CFS scores, there were several strategies used by stakeholders in each network to ensure timely and accurate assessments of frailty. Common strategies included: providing FPs with lists of the patients they were to assess for convenience; meeting with FPs in person or via email and/or phone bi-weekly or monthly; building on existing relationships with FPs; and attending FP meetings to explain expectations. Key stakeholders involved in facilitating data collection included: study co-investigators from each province, research assistants and coordinators, medical office assistants, data managers, and physician participants who supported other physicians within their clinics. In Alberta, having the Department Head support the project at the departmental level meant that recruitment was trivial, and collection was enabled by using an existing clinical support staff member who was able to remove nearly all barriers to data collection.

**Table 2:** Strategies used in working with FPs

Network (*n* = FPs recruited)	Strategies used in working with FPs to complete CFS	Stakeholders involved in strategies
British Columbia (*n* = 5)	∙ Provided complete lists of eligible patients to FPs; FPs determined which patients he/she was most comfortable in providing accurate assessments ∙ Research Assistant met with FPs in person to provide instructions and answer any questions ∙ Reminders and follow-ups via email and in-person bi-weekly	Co-investigator Research assistant Medical office assistants
Alberta (*n* = 52)	∙ Provided lists of 15 eligible patients to FPs ∙ Department of Family Medicine administrative personnel was associated with the clinic and thus provided continuous reminders to facilitate data collection ∙ Reminders and follow-ups were unnecessary; a research team member dropped into clinics during FPs’ spare time with lists of patients that needed to be assessed	Co-investigator Department of Family Medicine (Head of department and administrative personnel)
Manitoba (*n* = 10)	∙ FPs were familiar with preparing a search query of their patients who met eligibility criteria using the EMR ∙ Physicians at each site supported other physicians at their clinic by preparing a search query for them to review ∙ Reminders and follow-ups via email every 4-6 weeks. A phone call was arranged at the beginning of each site’s activity to confirm the query content and discuss approach with each participating physician.	Co-investigator Research coordinator Physician participants
Nova Scotia (*n* = 5)	∙ Mailed out packages with all required information, including lists of 100 eligible patients to FPs ∙ Reminders and follow-ups via email bi-weekly	Co-investigator Data manager
Ontario (*n* = 37)	∙ Discussion to assess interest and discuss value of study to family practice prior to network agreeing to study participation ∙ Hand-delivered complete lists of eligible patients to FPs extracted from MUSIC dataset; FPs determined which patients he/she was most comfortable in providing accurate assessments ∙ Co-I attended usual practice meetings with FPs and clinic management staff to explain study and study processes. ∙ Initial ask was timed to avoid other major local shifts in models of care delivery that took time and attention, and would have made this project burden and de-prioritised attention to project. ∙ Reminders and follow-ups in-person monthly	Co-investigator Research assistant

### Binary categorization of national demographic data

Table 3 displays demographic data for patients assigned a CFS score separated by their frail or not frail status. There was a total of 5466 CFS scores provided by clinicians across the five CPCSSN PBRLNs. About 18% of those assessed were identified as frail with a CFS score between 5 and 9. Percentages of frail (11-17%) and not frail patients (82-91%) were similar in all networks, with the exception of the MUSIC PBRLN in ON, who had a higher number of frail patients (25%) relative to the other networks. This finding is likely related to approximately half of clinicians providing care to deprived geographical regions in ON. These percentages of frailty are similar to previously reported frailty prevalence levels [23, 37–39]. The mean age was 81 for frail patients and 74 for those who were not frail. The majority of those identified as frail were female (65%) and had a significantly higher prevalence of hypertension, dementia, and depression than those who were not frail. Frail patients also had a greater number of medications prescribed and number of healthcare counters relative to non-frail patients. These findings align with other research examining frailty risk factors and outcomes [38–40].

**Table 3:** Binary Categorization of National Demographic Data (*N* = 5466)

Characteristic	Not Frail (CFS 1-4), N (%)	Frail (CFS 5-9), N (%)	*P*-value
N (%)	4460 (81.6)	1006 (18.4)	
Categorization by Province			
British Columbia	988 (86.9)	149 (13.1)	< 0.001*
Alberta	708 (82.5)	150 (17.5)	
Manitoba	779 (88.0)	106 (12.0)	
Ontario	1745 (75.4)	570 (24.6)	
Nova Scotia	240 (91.6)	31 (11.4)	
Demographics			
Age, mean (SD)	73.64 (6.6)	80.67 (8.7)	< 0.001†
Male	2077 (46.6)	348 (34.6)	< 0.001*
Frailty Risk Factors			
Missing Frailty Risk Factors	680 (15.2)	52 (5.2)	< 0.001*
Hypertension	2854 (75.5)	760 (79.7)	0.008*
Diabetes	1492 (39.5)	374 (39.2)	0.909*
Dementia	211 (5.6)	238 (24.9)	< 0.001*
Depression	839 (22.2)	316 (33.1)	< 0.001*
Osteoarthritis	1748 (46.2)	439 (46.0)	0.929*
Epilepsy	70 (1.9)	24 (2.5)	0.237*
BMI, mean (SD)	29.47 (6.4)	29.46 (6.9)	0.975†
Number of Medications (mean (SD))	6.84 (5.4)	10.48 (7.4)	< 0.001†
Number of Encounters in the Last Year (mean (SD))	6.60 (5.8)	8.71 (6.9)	< 0.001†

^*^P-value calculated using Chi-Squared tests

^†^*P*-value calculated using t-tests

### Categorization of national demographic data by severity of frailty

Table 4 displays demographic data for patients assigned a CFS score separated by frailty severity. When the 5466 patients with CFS scores were categorized according to severity of frailty, 17% were considered vulnerable (CFS score of 1-3), 15% were mildly to moderately frail (CFS score 50-6), and 3% were severely frail (CFS score 7-9). Vulnerable and mild-moderately frail patients are often considered the ideal patients that will benefit from intervention [41]. The constructs of older age, dementia, depression, and a greater number of prescribed medications represented a greater likelihood of frailty presence, as rated by FPs. These findings align with previously reported findings regarding frailty risk factors [38–40]. There was no obvious pattern between being diagnosed with hypertension, diabetes, osteoarthritis, epilepsy, increasing BMI, or number of healthcare encounters in relation to increasing levels of frailty.

**Table 4:** Categorization of National Demographic Data (*N* = 5466) by Severity of Frailty

Characteristic	Not frail (CFS 1-3)	Vulnerable (CFS 4)	Mildly to Moderately Frail (CFS 5-6)	Severely Frail (CFS 7-9)	*P*-value
N (%)	3520 (64.4)	940 (17.2)	839 (15.4)	167 (3.1)	
Categorization by Province					
British Columbia n(%); *N* = 1137	850 (74.8)	138 (12.1)	121 (10.6)	28 (2.5)	< 0.001*
Alberta n(%); *N* = 858	563 (65.6)	145 (16.9)	122 (14.2)	28 (3.3)	
Manitoba n(%); *N* = 885	585 (66.1)	194 (21.9)	97 (11.0)	9 (1.0)	
Ontario n(%); *N* = 2315	1319 (57.0)	426 (18.4)	477 (20.6)	93 (4.0)	
Nova Scotia n(%); *N* = 271	203 (74.9)	37 (13.7)	22 (8.1)	9 (3.3)	
Demographics					
Age, mean (SD))	73.0 (6.3)	76.1 (7.3)	80.1 (8.5)	83.5 (9.1)	< 0.001†
Male, N (%)	1681 (47.8)	396 (42.1)	288 (34.3)	60 (35.9)	< 0.001*
Frailty Risk Factors					
Missing Frailty Risk Factors	618 (17.6)	62 (6.6)	43 (5.1)	9 (5.4)	< 0.001*
Hypertension, N (%)	2153 (74.2)	701 (79.8)	637 (80.0)	123 (77.8)	< 0.001*
Diabetes, N (%)	1098 (37.8)	394 (44.9)	309 (38.8)	65 (41.1)	0.003*
Dementia, N (%)	135 (4.7)	76 (8.7)	166 (20.9)	72 (45.6)	< 0.001*
Depression, N (%)	585 (20.2)	254 (28.9)	269 (33.8)	47 (29.7)	< 0.001*
Osteoarthritis, N (%)	1309 (45.1)	439 (50.0)	376 (47.2)	63 (39.9)	0.025*
Epilepsy, N (%)	57 (2.0)	13 (1.5)	17 (2.1)	7 (4.4)	0.107*
BMI, mean (SD)	29.0 (6.0)	31.5 (7.8)	29.6 (7.0)	28.3 (6.2)	< 0.001†
Number of Medications (mean (SD))	6.4 (4.9)	8.6 (6.5)	10.3 (7.3)	11.4 (7.5)	< 0.001†
Number of Encounters in the Last Year (mean (SD))	6.1 (5.3)	8.4 (6.9)	8.8 (6.8)	8.1 (7.2)	< 0.001†

^*^P-value calculated using Chi-Squared tests

^†^*P*-value calculated using t-tests

### Frailty by province

Table 5 displays demographic data for patients assigned a CFS score separated by province. There were 1006 patients (18%) who were identified as frail across the 5 provinces. There was a statistically significant difference (*p* < 0.05) between the 5 provinces for all frailty risk factors with the exception of diagnoses of hypertension and dementia. The average age of frail individuals ranged from 79 to 84; the average percentage of frail patients who are female ranged from 56 to 71%. These findings align with previous research indicating that frailty risk increases with age and is more likely to occur in females [39, 42].

**Table 5:** Frailty (CFS 5-9) by Province

Characteristic	Alberta	British Columbia	Manitoba	Nova Scotia	Ontario	*p*-value
N	150	149	106	31	570	
Demographics						
Age, mean (SD)	78.5 (8.4)	84.7 (8.0)	79.9 (7.9)	83.6 (9.6)	80.2 (8.7)	< 0.001†
Male, N (%)	43 (28.7)	60 (40.3)	47 (44.3)	9 (29.0)	189 (33.2)	0.043*
Frailty Risk Factors						
Missing Frailty Risk Factors	8 (5.3)	8 (5.4)	4 (3.8)	2 (6.5)	30 (5.3)	0.968*
Hypertension, N (%)	114 (80.3)	119 (84.4)	82 (80.4)	26 (89.7)	419 (77.6)	0.264*
Diabetes, N (%)	84 (59.2)	43 (30.5)	40 (39.2)	9 (31.0)	198 (36.7)	< 0.001*
Dementia, N (%)	33 (23.2)	46 (32.6)	26 (25.5)	9 (31.0)	124 (23.0)	0.172*
Depression, N (%)	56 (39.4)	43 (30.5)	14 (13.7)	12 (41.4)	191 (35.4)	< 0.001*
Osteoarthritis, N (%)	71 (50.0)	45 (31.9)	90 (88.2)	8 (27.6)	225 (41.7)	< 0.001*
Epilepsy, N (%)	3 (2.1)	14 (9.9)	1 (1.0)	0 (0.0)	6 (1.1)	< 0.001*
BMI, mean (SD)	30.8 (7.8)	26.7 (4.4)	30.3 (6.9)	29.2 (9.5)	29.3 (6.8)	0.006†
Number of Medications (mean (SD))	12.8 (8.4)	6.8 (4.5)	3.9 (4.6)	7.2 (4.5)	12.2 (7.1)	< 0.001†
Number of Encounters in the Last Year (mean (SD))	8.1 (5.7)	11.0 (8.0)	10.8 (8.3)	2.9 (2.4)	8.2 (6.4)	< 0.001†

^*^P-value calculated using Chi-Squared tests

^†^*P*-value calculated using t-tests

### Key stakeholders

Table 6 displays numbers of several key stakeholders involved in the research process, including researchers and co-investigators, FPs, data managers, research assistants/coordinators/students, and clinic staff. Numbers of stakeholders varied considerably across the provinces from 2 to 11, nonexclusive of clinician participants.

**Table 6:** Key Stakeholders in each Network

Stakeholder(s)	British Columbia	Alberta	Manitoba	Nova Scotia	Ontario
Researchers/Co-Is	3	4	2	1	1
Clinicians (FPs)	5	52	10	5	37
Data Managers	2	1	1	1	1
Research Assistants/Coordinator & Students	1	2	1	0	1
Clinic Staff	6	2	3	0	1
Total including FPs	17	61	17	7	41
Total without FPs	11	9	7	2	4

## Summary of research process informed by the CFIR

### Intervention characteristics

Some FPs received a random sample of their patients (AB, NS) and provided a CFS score for each patient on their list. Others received a complete list of their patient population who met the eligibility criteria (BC, ON, MB) and provided scores for those patients they were most familiar with and felt most confident assigning accurate scores for. In all networks, FPs had the option of using their EMR records to gather information to assign a CFS score; however, most were comfortable using recall as they were quite familiar with their patients. All networks reported that the FPs were familiar with the CFS tool, but support was available if they had questions.

To facilitate data collection, common strategies were used across the networks, including in-person, telephone, and email communication. In BC, a research assistant was in constant communication with FPs; meetings were scheduled during two of the FPs’ lunch hours and sometimes outside of work hours in which the research assistant supported the recording of CFS scores. In AB, a research team member who was also clinic staff personnel facilitated data collection for each of the FPs. In all networks, research team members (coordinators, administrative staff, students) maintained contact with the physicians and clinic staff to ensure that data collection was complete.

### Outer setting

This work did not directly involve patients. The work was completed with individual FPs in primary healthcare clinics and thus there was no networking with external organizations by the FPs. Future utility to patient and primary care was part of the initial engagement discussion with FPs and key to participation.

Although competing organizations were not an issue within this work, there was a need for peer pressure *across* the various networks. For example, efforts were made to adhere to self imposed project timelines. Yet, multiple reminders were often needed from the project coordinator to separate networks and to all networks so they could see how their colleagues were doing with regards to completing the task. There was also a sense of pressure reported by NS when other networks collected more data, but NS had a lower than expected uptake.

Networks reported feeling frustration with delays in procedures such as ethics approval as the main barrier to data collection. NS and ON faced several delays with ethics resulting in competing pressure for participating FPs’ attention at meetings where the CFS data collection was to be initiated. Specifically, in ON these meetings (Ontario Health Teams Initiative from the ON Ministry of Health) had major implications for primary care policy and generated intense activity at the time of planned data collection. A decision was made to delay initiation until there was time without pressure from this, and while this resulted in delay in collecting data (about which the network felt pressure), this paid off in subsequent high and enthusiastic uptake by FPs. Furthermore, the ON network was added much later to the project than other networks and thus had to catch up with study planning and protocol. These delays resulted in the research team as a whole unable to adhere to the funders’ deadlines, which had a negative effect on funding decisions.

Additionally, no incentives were provided to physicians in any of the networks with the exception of MB, in which monetary incentives were provided after completion of assessments ($2.50 for each assessment score). For the most part, FPs were motivated by the potential value of this work to primary care.

### Inner setting

Although FPs saw the relevance and importance of the work and were interested, it was challenging for them to prioritize engagement and commitment to implementation without additional support. The time burden for FPs of day-to-day patient care is already high. And although the CFS is a low budget tool requiring minimal time, this task was one of many for FPs amidst primary care transformations and immediate clinical tasks. FPs have limited time to devote to clinical tasks and even less time for data recording tasks that do not relate to clinical workflow. It is estimated that, on average, a FP spends 1-2 h of after-clinic time each day attending to administrative tasks [43, 44]. Immediate tasks that impact patient care and workflow supporting this are necessarily prioritised over work that is not remunerated.

Engagement within CPCSSN PBRLNs was generally not an issue and physician-to-physician sharing about the ease of the work led to additional FPs willing to participate. There was potential for high extrinsic incentives related to this work for all networks such as increased stature, goal sharing, changing practice related to frailty identification and management, and potential for publications and knowledge dissemination activities. FPs also saw the value of other quality improvement initiatives they might want to initiate, which was key to engagement.

### Characteristics of individuals

CPCSSN PBRLN directors facilitated this work within each of their networks; however, students, research coordinators, and administrative staff were key to this process. All research team members were invested in the work and maintained consistent communication with the team through regular team meetings and email communication, as well as with clinics and FPs. The research team took steps to create a climate of trust and respect for FPs’ involvement in the study, while building and maintaining relationships with clinic staff. Communication lines were always left open for feedback and questions throughout the study, and FPs and clinic staff had the contact information of researchers available to them at all times. Research assistants and network leaders also provided multiple reminders and check-ins with FPs either in person or via email in order to ensure timely data collection. In BC, a research assistant met with FPs in person to answer questions and to assist with data recording. In ON, the network leader attended FPs usual practice meetings to explain the study and study processes.

### Process of implementation

The general plan for this work was created in collaboration between research team members. The implementation of the data collection process and using the CFS was created within each CPCSSN PBRLN. Relationships were key in all networks, leading to snowball sampling and some physicians assisting and motivating others within their clinics (ON, BC). Understanding and discussing the relevance of the project and frailty in a broader sense was key in maintaining these relationships. In BC, one physician was considered a champion. He dedicated additional time and effort to help with this work, including suggesting other FPs who would be willing to be involved, and participating in webinars.

Regular team meetings involving research team members were essential to move the project along and to ensure alignment of procedures. The amount of time taken to collect data and then extract EMR data to be compiled into a pan-Canadian dataset varied considerably by network, ranging from 6 weeks to 10 months from the time of ethical approval. The final dataset was compiled in January 2020.

## Discussion: key challenges & lessons learned

There were several noteworthy challenges experienced throughout the research process related to differences across provinces, specifically in the areas of: ethics applications, team meetings, trade-offs in engagement of FPs, the need for structural support, and data sharing.

CPCSSN promotes multi-jurisdictional research that allows for the exploration of these differences as well as how to potentially reduce variation across systems. Multi-jurisdictional PBRLNs bring together professionals from various disciplines to study the organization, financing, and delivery of health care resources in real-world practice settings, with the goal of producing actionable evidence to improve practice and national policies [45].

### Ethics applications

Adhering to timelines was a key challenge that ultimately affected funding decisions by the CFN. Networks worked off of one another’s ethics applications. Each network individually applied for ethics approval for the work, which created a challenge for the overall study timeline. Ethics approval timelines were not aligned and thus data collection was extremely delayed in both NS and ON (ON faced the additional challenge of joining the research team later relative to other network), resulting in a subsequent delay in national data compilation and analysis. NS was also required to re-apply for ethics due to changes in physicians’ EMR systems. Ethical oversight related challenges are reported as barriers to PBRLN work [11]; we suggest mapping ethics board timelines and having standard block text for components of ethics applications to ensure all team members are clear on the detail and processes of the study. A key lesson learned from these challenges is the need for a protocol for all team members to follow that allows the work to be completed but also allows for a flexible process. Each network was required to tailor the data collection protocol to fit with their FPs’ worldviews. Dania et al. [11] report a significant barrier to PBRLN work as being lengthened timelines. Ethical approval across networks is time-consuming. We suggest a six-month lead time for networks to get through ethics and work out study details and protocols.

### Team meetings

Overall, team meetings were successful and disagreements across provincial stakeholders were settled through discussion and consensus. However, collaboration between CPCSSN network leaders, co-investigators, and other research team members was at times challenging. Campbell-Voytal et al.’s [10] study involving seven PBRLNs working in collaborative teams to articulate procedures for PBRN Research Good Practices reports the importance of getting through rocky periods with trust, communication, and handling conflict as key factors to successful PBRLN collaboration. Part of multi-jurisdictional work involves working with differing time zones, competing demands of all those involved, and the increased complexity of research coordination [11]. Efforts were made to ensure that as many team members as possible could attend these meetings; however, this was not always possible. And although the project coordinator recorded meeting minutes and distributed them to the team, there was a risk of team members failing to review these documents, potentially leading to missed information. Regular communication between team members is a key factor for successful research [46, 47] but has been reported as a barrier to PBRLN work due to increased efforts. Dania et al. [11] report a risk for communication and understanding gaps in large networks during research activities, further exacerbated by distance communication. Thus, a key lesson learned for working across networks was to make all possible efforts to maintain communication through the prioritization of regular team meetings with all team members to ensure everyone is aligned in study procedures and timelines.

### Trade-offs in engagement of FPs

PBRLNs contribute to knowledge production and utilization and serve as learning communities and drivers of quality improvement [48]. Data on the processes of primary health care are essential to inform decision making at the practice and system level to enable primary health care research [48].

PBRLNs aim to stimulate the development of research that reflects the challenges and context of primary health care practice [48]. Dania et al. [11] report common barriers to engagement in research being a lack of time, low interest in research topics, limited research skills, and competing priorities. In our study, there were differences related to FP’s level of engagement with the work and the assessments they provided. There was no direct incentive back to individuals, which may have resulted in less willingness to participate. Due to competing demands, FPs generally had a low level of motivation to participate, perhaps influencing the accuracy of their assessments. Fagnan et al.’s [49] qualitative study reports that motivation for participation in practice-based research includes personal satisfaction, improving local clinic-based care, and contributing to community and system level improvements. Additionally, Dania et al. [11] report that research engagement is enhanced when practitioners are involved in initiating, designing, and driving research. It is possible that engaging FPs earlier in the research process and involving them in team meetings might have helped facilitate a greater interest.

Conducting studies across networks requires leadership from network directors and research team members. Relationships with FPs proved to be a key factor in navigating the study setting and obtaining timely data, including continuous follow-up and checking in with progress. Clear communication with staff in each network was key, as well as 1:1 follow-up emails to drive the process forward. Early engagement between research team members and stakeholders and meaningful relationships are key to promoting active involvement in and successful uptake of context specific research [47, 50–52]. Two key lessons learned from these challenges are the need for a laddering of incentives [11] to get through data collection and having a focused timeline across networks to get the job done.

FPs may have also differed in whether they used only recall to provide their assessments, or whether they accessed additional records, thus influencing accuracy of assessment scores. Additionally, some FPs were provided with a complete list of their patients, which was sometimes greater than 500. Although FPs were told they did not need to score all of these patients, but could rather choose those who they felt most confident providing accurate scores for, this process was likely more overwhelming compared to receiving a list of 15 patients to assess (as in AB). Thus, there was a higher intensity of workload required for FPs who needed to provide a greater number of CFS scores vs. a lower intensity for FPs required to provide fewer CFS scores. Recruiting a greater number of FPs in some networks to more evenly distribute patients may have helped reduce this burden. Another notable consideration is the differing numbers of stakeholders involved in each network, ranging from 2 to 11, potentially influencing the burden of tasks within each network and ability to adhere to study timelines.

### Need for structural support

In AB, the process of CFS implementation was supported by structure. The project aligned with the University of Calgary’s Department of Family Medicine’s goals and values and thus they were able to provide additional support. Alignment of values between stakeholders and researchers in terms of a common purpose and goal is essential in increasing the relevance, meaning, and uptake of the research [53]. The department provided temporary staff to facilitate data collection, data extraction, and data linking. FPs in the department were motivated to complete data collection because this research became a part of their everyday work and they had developed good relationships with team members. Administrative staff from the department were also associated with the clinics and were able to easily gain entry to provide continuous reminders to facilitate data collection. Campbell-Voytal et al. [10] report a key lesson learned in their work with PBRLNs; they found participants involved in a study relevant to their needs and interests will engage and persist despite multiple barriers. Dania et al. [11] also report motivation to participate in research being associated with the presence of a research culture. Similar support and motivation that was evident in AB in other provinces may have made the process of CFS implementation smoother.

### Data sharing

One of the key challenges of working across networks is the difference in EMR systems and data that can be used for analysis. Research data quality pertaining to recording, documentation, and coding, and a lack of uniform terminology are reported batters to PBRLN work [11].

Interoperability of EMR systems is also a known challenge to conducting research in different contexts [54–57]. Often, specific data needs to be excluded when there is a lack of consistency across the various networks. Thus, a key difference among networks that may have potentially influenced study analysis and results was related to using only common data. This study relied on physician documentation in the EMR. Processing algorithms and unique EMR characteristics may have prevented retrieval of some patient characteristics. For example, if assessments related to a functional test for frailty were present within one network’s EMR system, but not in other networks, this data could not be used for national analysis. Free-text data was also not available from all provinces, thus free-text data was not able to be incorporated; the same applied with a large number of blood-work related test results (only hemoglobin A1C, creatinine and estimated glomerular filtration rate was able to be included). In Manitoba, depression and number of unique medications may have been entered into the EMRs differently by physicians, reflecting the differences observed in the number of unique medications prescribed and proportion of older adults with depression relative to the other CPCSSN sites.

## Conclusion

The differences noted across CPCSSN networks in our frailty study highlight the benefits and challenges of multi-jurisdictional work across provinces. Ultimately, differences in ability to adhere to timelines, different competing pressures in the outer settings in different jurisdictions, numbers of stakeholders present in each network to share study tasks, local ethics timelines and processes, level of engagement with the research, differences in structural support, and systematic differences in data collection and EMR use influenced our study processes and output. If PBRLNs are going to be successful in sharing the common interest of improving quality of healthcare, as much alignment regarding goals of activities and work as possible is needed. Various processes across PBRLNs make for an engaged and rich but challenging environment. It is essential to prioritize continuity and congruence when collaborating with stakeholders across multiple jurisdictions through ongoing communication, clear goals, and trusting relationships in order to improve health and healthcare delivery.

## Figures and Tables

**Fig. 1:**
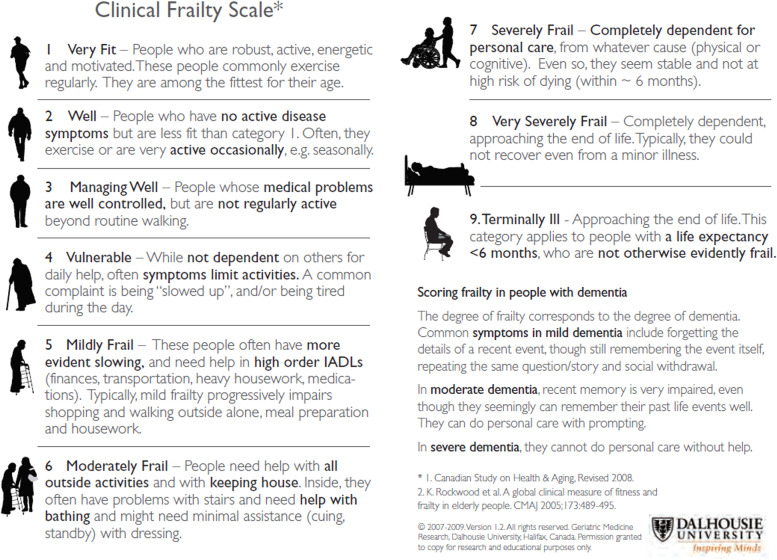
Rockwood Clinical Frailty Scale. Rockwood, K., Song, X., MacKnight, C., Bergman, H., Hogan, D. B., McDowell, I., & Mitnitski, A. (2005). A global clinical measure of fitness and frailty in elderly people. Cmaj, 173(5), 489-495. https://doi.org/10.1503/cmaj.050051

**Fig. 2:**
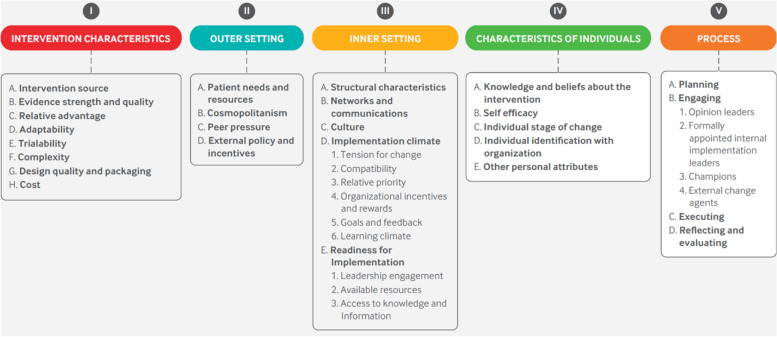
Consolidated Framework for Implementation Research. Nolan, M. B., & Warner, D. O. (2017). Perioperative tobacco use treatments: putting them into practice. Bmj, 358, j3340. https://doi.org/10.1136/bmj.j3340

## Data Availability

All data generated or analysed during this study are included in this published article.

## Appendix 1

Fig. 1

## Appendix 2

Fig. 2

## Abbreviations

EMR: Electronic medical record
LHS: Learning health system
PBRLN: Practice-based research and learning network
CPCSSN: Canadian Primary Care Sentinel Surveillance Network
BC: British Columbia
AB: Alberta
SAPCReN: Southern Alberta Primary Care Research Network
MB: Manitoba
ON: Ontario
MUSIC: McMaster University Sentinel and Information Collaboration
NS: Nova Scotia
CFS: Clinical Frailty Scale
CGA: Comprehensive Geriatric Assessment
CFIR: Consolidated Framework for Implementation Research
FP: Family physician
